# Acinar cystic transformation of the pancreas causing progressive main pancreatic duct dilation: a diagnostic dilemma

**DOI:** 10.1093/jscr/rjad154

**Published:** 2023-03-28

**Authors:** Elizabeth Sebastian, Anthony Longano, Matthew Y K Wei, Janindu Goonawardena, Nicholas Bull, Sayed Hassen

**Affiliations:** Department of Upper GI Surgery, Eastern Health, Melbourne, Victoria, Australia; Department of Pathology, Eastern Health, Melbourne, Australia; Department of Upper GI Surgery, Eastern Health, Melbourne, Victoria, Australia; Department of Upper GI Surgery, Eastern Health, Melbourne, Victoria, Australia; Department of Upper GI Surgery, Eastern Health, Melbourne, Victoria, Australia; Department of Upper GI Surgery, Eastern Health, Melbourne, Victoria, Australia

## Abstract

Acinar cystic transformation (ACT) of the pancreas is a rare benign lesion. We describe a case of ACT with progressive main pancreatic duct dilation concerning for malignancy, not previously described. We discuss the difficulties associated with imaging and biopsy in differentiating this pathology from other cystic lesions, including intraductal mucinous papillary neoplasms.

## INTRODUCTION

Acinar cystic transformation (ACT) of the pancreas, also known as acinar cell cystadenoma, is a very rare, benign cystic lesion with acinar differentiation of the cyst lining demonstrated on microscopy [1]. Recent literature suggests that it is a non-neoplastic lesion associated with progressive ballooning of the acinar epithelium [2]. Predominantly found in women, they can occur as unilocular or multilocular lesions and are generally solitary in nature [3]. We report a case of ACT with progressive pancreatic duct dilation masquerading as mixed-type intraductal mucinous papillary neoplasms (IPMN).

## CASE REPORT

A 51-year-old female had a staging computed tomography (CT) scan for anal squamous cell carcinoma, previously treated with definitive chemoradiotherapy. A large cystic lesion in the head of the pancreas was detected incidentally. Additional background medical history included chronic alcoholic pancreatitis and insulin dependent diabetes mellitus. There was a family history of pancreatic adenocarcinoma in her paternal grandfather. Abdominal examination was unremarkable. Blood tests revealed a normal lipase and bilirubin with a mild elevation of liver enzymes (alkaline phosphatase  155 μmol/L; gamma-glutamyl transferase 198 μmol/L).

**Figure 1:**
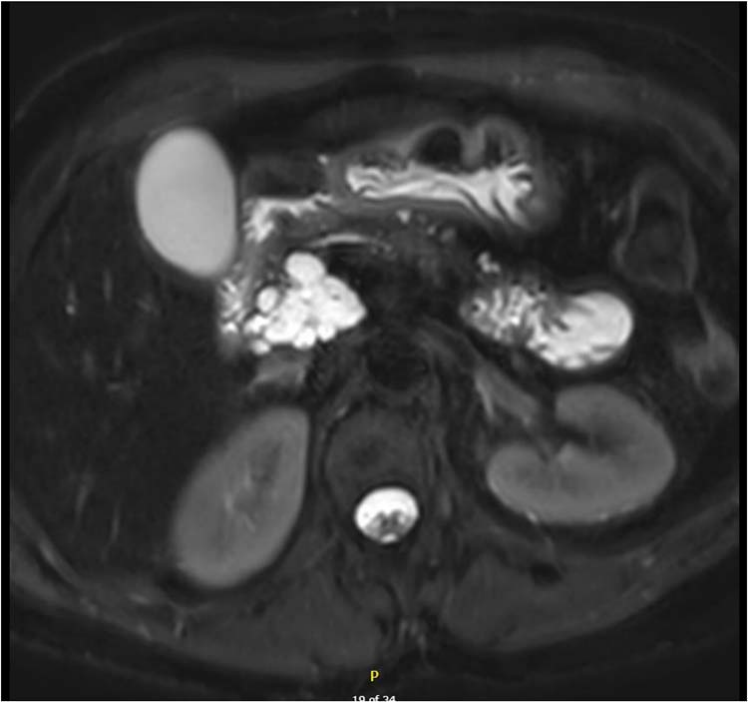
Multi-loculated head of pancreas cyst, with no pancreatic duct dilation.

Magnetic resonance imaging (MRI) revealed a 6.9 cm multi-lobulated cystic pancreatic head lesion with underlying background changes of alcohol-related chronic pancreatitis. There was no biliary or pancreatic duct dilation, or other suspicious features for malignancy (Fig. 1). This lesion was thought to be a serous cystadenoma or pancreatic pseudocyst. Endoscopic ultrasound (EUS) revealed innumerable small cysts consistent with serous cystadenoma and fine-needle biopsy demonstrated benign ductal epithelium.

**Figure 2:**
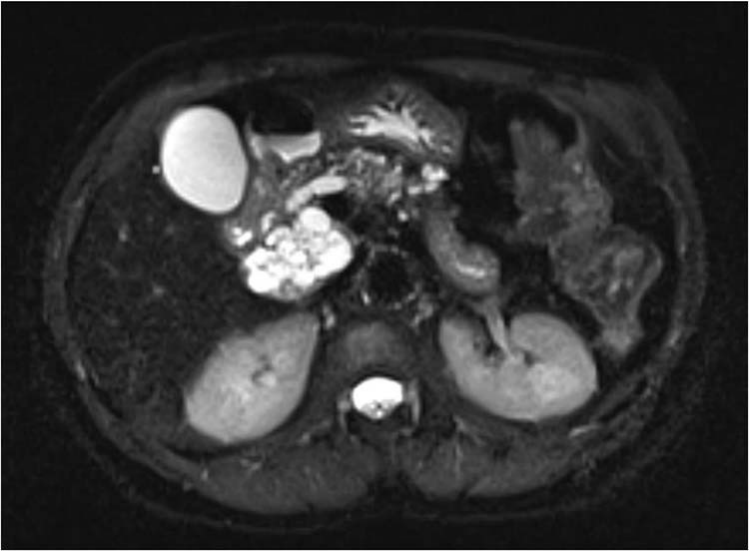
Increased diameter of lesion and dilation of main pancreatic duct on interval MRI.

Interval MRI after 1 year showed an increased size of 8 cm with associated main pancreatic duct dilation up to 8 mm, which was not previously present (Fig. 2). Repeat EUS and biopsy demonstrated atypical cytology. The presence of atypical cytology and progressive duct dilation suggested the possibility of a malignant process and surgical resection was recommended.

After extensive patient discussion a Whipple procedure (pancreaticoduodenectomy) was performed. A cystic pancreatic head lesion was present intraoperatively. The remainder of the pancreas had a firm consistency in line with the history of previous pancreatitis. Post-operative recovery was uneventful, and she was discharged home after 14 days.

Macroscopically the lesion was 85 × 42 × 35 mm in size, white in colour with a multi-loculated cystic structure. The septa between cysts were heavily calcified. The lesion did not appear to arise from the main pancreatic duct but was confined to the head of the pancreas and clear of margins. In total, 10 benign peri-pancreatic lymph nodes were identified.

**Figure 3:**
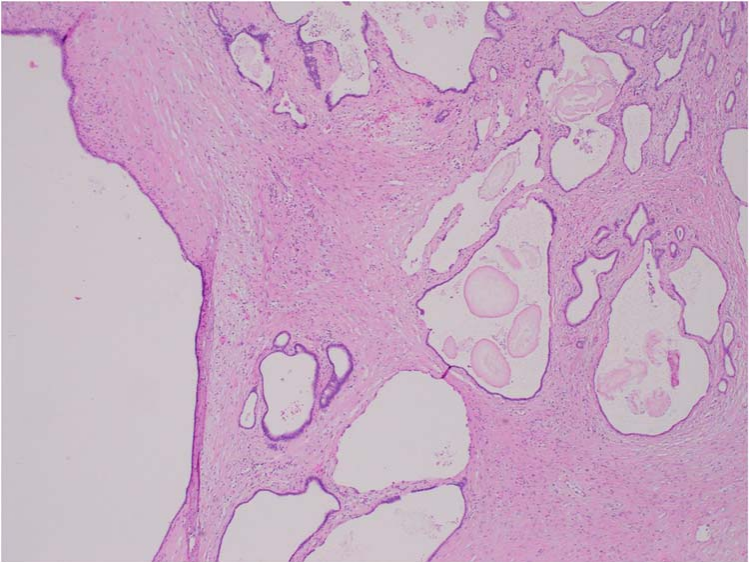
Hematoxylin and Eosin (H&E) × 40 showing benign cysts and luminal eosinophilic concretions.

**Figure 4:**
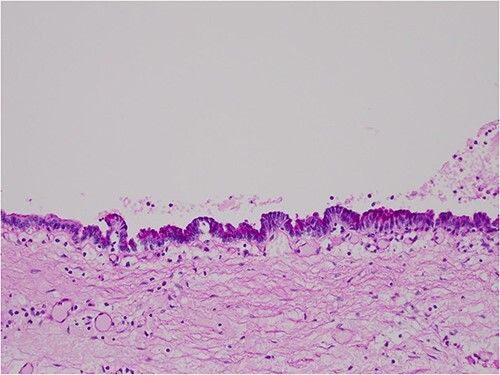
PASD × 200 showing positive apical staining in cyst-lining epithelial cells, indicating acinar differentiation.

The histologic examination revealed numerous large, medium and small cysts separated by prominent stromal areas of fibrosis. These areas contained luminal eosinophilic concretions (Fig. 3). No ovarian-type parenchyma was found to suggest IPMN. The cyst formation maintained the outline of the native lobules, with some withered central islets appreciated. No papillary architecture was observed, and no epithelial dysplasia or invasive carcinoma was present. Epithelium lining the cysts was attenuated in some areas, and cuboidal in others. Where the epithelium was recognizably cuboidal, there was scattered acinar differentiation noted, characterized by apical cytoplasmic granules positive on periodic acid-Schiff-diastase (PASD) staining (Fig. 4). Acinar differentiation did not therefore have to be confirmed with further immunohistochemistry.

Based on these histological findings the final diagnosis was ACT of the pancreas. After discussion in a multidisciplinary meeting the patient was recommended for clinical follow-up. Written consent was obtained from the patient for this report.

## DISCUSSION

ACT of the pancreas was described in 2002 by Albores-Saavedra as acinar cystadenoma [4]. The 2019 World Health Organization updated classification of tumours of the digestive system recognized its non-neoplastic nature through molecular clonality analysis, and thus completely distinct from acinar cell carcinoma or acinar cell cystadenocarcinoma [2, 5].

No clear guidelines for pre-operative diagnosis of ACT exist. It most commonly presents with abdominal pain but s often found incidentally, as with other cystic pancreatic lesions, on abdominal imaging for other indications [3]. It occurs more commonly in females but other clinical risk factors are not well understood. ACT can involve any part or the entirety of the pancreas and surgical resection may be a Whipple procedure, distal pancreatectomy or total pancreatectomy. There is no clear surveillance protocol, but there have been no reported cases of malignant transformation associated with ACT [3].

Pre-operative fine-needle aspiration biopsy via EUS can be useful, however histopathological analysis is often inaccurate due to sampling errors and difficulty preserving the tissue architecture that demonstrates acinar cell differentiation [6]. Further developments in EUS tissue acquisition and immunohistochemistry or genetic analysis of biopsy specimens could provide significant improvements in pre-operative assessment [3]. Definitive diagnosis of ACT is, therefore, generally obtained on post-operative histopathology.

A low-powered retrospective study of radiological features differentiating ACT from side-branch IPMN identified four diagnostic criteria, which in combination could suggest ACT. These were: five or more cysts; clustered peripheral small cysts; presence of cyst calcifications and absence of communication with the main pancreatic duct. Presence of three out of four criteria had a sensitivity of 80% and specificity of 100% for ACT [7]. In this case, three criteria were present. However, the presence of atypical cytology on EUS and interval dilation of the main pancreatic duct increased suspicion for a pre-malignant or malignant process and a pancreatic resection was required.

## CONCLUSION

ACT of the pancreas is a rare benign pathology that should be considered in patients with a pancreatic cystic lesion. Certain radiological findings can help distinguish ACT from other cystic lesions. However, there is a lack of reliable clinical and histopathological features to provide accurate pre-operative diagnosis for this condition. Our case demonstrates progressive pancreatic duct dilation in ACT mimicking mixed-type IPMN not previously described. Further research in pre-operative diagnosis of ACT could help avoid the significant morbidity of pancreatic resection for patients with this benign condition.

## Data Availability

All data underlying the results are available as part of the article and no additional source data are required.
